# From the Editor-in-Chief

**DOI:** 10.34763/jmotherandchild.2020241.1915.000005

**Published:** 2020-07-29

**Authors:** Jolanta Sykut-Cegielska

On behalf of myself and the Editorial Board of the *Journal of Mother and Child*, I am pleased to welcome all our Readers and Authors with the first issue of our *Journal*.

At the same time, I would like to inform you about the important changes that have taken place recently at our *Journal.*.

As you may have noticed, this year, we have changed the name of our *Journal*, and it is now called the *Journal of Mother and Child* (*JMC*). We will still retain the original Polish title, i.e. *Medycyna Wieku Rozwojowego*, in order to maintain an editorial continuity. Later this year, we also intend to archive the previous issues and make them available online on the new platform.

From 2020, the *JMC* will be published by a new publisher – Sciendo (De Gruyter Group), and soon, it will be available in an electronic version only, with only a few printed copies produced mainly for libraries. Authors will receive their papers online in a portable document format (pdf) form.

In order to cover all relevant medical disciplines, I have taken a decision to expand the *JMC* Editorial Board by inviting experts from respective fields to serve as Section Editors and International Editorial Scientific Board members. I would like to take this opportunity to thank all those fantastic people who have accepted the invitation and will be supporting us on our exciting journey.

As you may know, the *JMC*/*Medycyna Wieku Rozwojowego* has been published for more than 20 years as a quarterly journal and is now indexed in MEDLINE/PubMed, Scopus, Index Copernicus, EBSCO, BioMedLib, Research Gate, MNiSW and Polish Medical Bibliography. Our current target is to have the *JMC* indexed in other international indexing databases and catalogues, such as Web of Science.

All the above-mentioned changes and efforts are aimed at improving the quality of the journal's content while continuing its general concept and scope. The *Journal's* scope is based on a holistic approach towards a patient, including all medical aspects as well as other important life areas, such as nutrition, psychology and social or ethical issues. Most importantly, we focus on a patient through all stages of the human life cycle, from the early years, i.e. the prenatal and perinatal periods, through the developmental period, until adolescence and adulthood (maternal health). Moreover, the *Journal of Mother and Child* should be a platform for presenting the results from all areas of research studies that aim to improve human health, reflecting the connection between clinical knowledge and basic science, called translational medicine. I believe that such a broad approach makes us unique among existing medical journals.

My ambition as an Editor-in-Chief will be to include in the *Journal* not only articles related to common diseases and disorders, which are important from an epidemiological viewpoint, but also those describing rare conditions. Rare diseases require special attention for several reasons and have become an increasingly important topic in medical literature. Following this direction, the second issue this year will be dedicated exclusively to the category of rare inherited metabolic diseases and will be published as a Special Issue with invited papers from well-known experts of metabolic medicine. I believe that such an approach will make our *Journal* attractive to Authors, Reviewers and Readers.

Last but not the least, I would like to encourage both experienced and young researchers to submit their interesting original manuscripts, reviews and case reports to the *Journal of Mother and Child*, including your ideas for Special Issues.

